# Supplementary material for: Interplay between charge distribution and DNA in shaping HP1 paralog phase separation and localization
Source: eLife. 2024 Apr 9;12:RP90820. doi: 10.7554/eLife.90820 (PMC11003746; doi:10.7554/eLife.90820)
Supplement: Supplementary file 1. [file elife-90820-supp1.docx]

Supplementary File for

**Interplay between charge distribution and DNA in shaping HP1 paralog phase separation and localization**

**Tien M. Phan^1a^, Young C. Kim^2^, Galia T. Debelouchina^3*^, Jeetain Mittal^1a,b,c*^**

*^1a^Artie McFerrin Department of Chemical Engineering, Texas A&M University, College Station, TX, USA*

*^1b^Department of Chemistry, Texas A&M University, College Station, TX, USA*

*^1c^Interdisciplinary Graduate Program in Genetics and Genomics, Texas A&M University, College Station, TX, USA*

*^2^Center for Materials Physics and Technology, Naval Research Laboratory, Washington, DC, USA*

*^3^Department of Chemistry and Biochemistry, University of California San Diego, La Jolla, CA, USA*

*^*^Corresponding authors:* [*gdebelouchina@ucsd.edu*](mailto:gdebelouchina@ucsd.edu)*;* [*jeetain@tamu.edu*](mailto:jeetain@tamu.edu)

**a)** Sequences of HP1 paralogs and their chimeras. The CD and CSD domains are highlighted in red and blue, respectively.

| HP1α  (191aa) | MGKKTKRTADSSSSEDEEEYVVEKVLDRRVVKGQVEYLLKWKGFSEEHNTWEPEKNLDCPELISEFMKKYKKMKEGENNKPREKSESNKRKSNFSNSADDIKSKKKREQSNDIARGFERGLEPEKIIGATDSCGDLMFLMKWKDTDEADLVLAKEANVKCPQIVIAFYEERLTWHAYPEDAENKEKETAKS |
| --- | --- |
| HP1β  (185aa) | MGKKQNKKKVEEVLEEEEEEYVVEKVLDRRVVKGKVEYLLKWKGFSDEDNTWEPEENLDCPDLIAEFLQSQKTAHETDKSEGGKRKADSDSEDKGEESKPKKKKEESEKPRGFARGLEPERIIGATDSSGELMFLMKWKNSDEADLVPAKEANVKCPQVVISFYEERLTWHSYPSEDDDKKDDKN |
| HP1γ  (183aa) | MASNKTTLQKMGKKQNGKSKKVEEAEPEEFVVEKVLDRRVVNGKVEYFLKWKGFTDADNTWEPEENLDCPELIEAFLNSQKAGKEKDGTKRKSLSDSESDDSKSKKKRDAADKPRGFARGLDPERIIGATDSSGELMFLMKWKDSDEADLVLAKEANMKCPQIVIAFYEERLTWHSCPEDEAQ |
| HP1α-βIDRs  (185aa) | MGKKQNKKKVEEVLEEEEEEYVVEKVLDRRVVKGQVEYLLKWKGFSEEHNTWEPEKNLDCPELISEFMQSQKTAHETDKSEGGKRKADSDSEDKGEESKPKKKKEESEKPRGFERGLEPEKIIGATDSCGDLMFLMKWKDTDEADLVLAKEANVKCPQIVIAFYEERLTWHAYPSEDDDKKDDKN |
| HP1α-γIDRs  (179aa) | MASNKTTLQKMGKKQNGKSKKVEEAEPEEYVVEKVLDRRVVKGQVEYLLKWKGFSEEHNTWEPEKNLDCPELISEFMKAGKEKDGTKRKSLSDSESDDSKSKKKRDAADKPRGFERGLEPEKIIGATDSCGDLMFLMKWKDTDEADLVLAKEANVKCPQIVIAFYEERLTWHAYPEDEAQ |
| HP1α-βHinge  (186aa) | MGKKTKRTADSSSSEDEEEYVVEKVLDRRVVKGQVEYLLKWKGFSEEHNTWEPEKNLDCPELISEFMQSQKTAHETDKSEGGKRKADSDSEDKGEESKPKKKKEESEKPRGFERGLEPEKIIGATDSCGDLMFLMKWKDTDEADLVLAKEANVKCPQIVIAFYEERLTWHAYPEDAENKEKETAKS |
| HP1α-γHinge  (178aa) | MGKKTKRTADSSSSEDEEEYVVEKVLDRRVVKGQVEYLLKWKGFSEEHNTWEPEKNLDCPELISEFMKAGKEKDGTKRKSLSDSESDDSKSKKKRDAADKPRGFERGLEPEKIIGATDSCGDLMFLMKWKDTDEADLVLAKEANVKCPQIVIAFYEERLTWHAYPEDAENKEKETAKS |
| HP1β-αHinge  (190aa) | MGKKQNKKKVEEVLEEEEEEYVVEKVLDRRVVKGKVEYLLKWKGFSDEDNTWEPEENLDCPDLIAEFLKKYKKMKEGENNKPREKSESNKRKSNFSNSADDIKSKKKREQSNDIARGFARGLEPERIIGATDSSGELMFLMKWKNSDEADLVPAKEANVKCPQVVISFYEERLTWHSYPSEDDDKKDDKN |
| HP1γ-αHinge  (196aa) | MASNKTTLQKMGKKQNGKSKKVEEAEPEEFVVEKVLDRRVVNGKVEYFLKWKGFTDADNTWEPEENLDCPELIEAFLNSQKKYKKMKEGENNKPREKSESNKRKSNFSNSADDIKSKKKREQSNDIARGFARGLDPERIIGATDSSGELMFLMKWKDSDEADLVLAKEANMKCPQIVIAFYEERLTWHSCPEDEAQ |
| HP1α-βCDβCSD  (190aa) | MGKKTKRTADSSSSEDEEEYVVEKVLDRRVVKGKVEYLLKWKGFSDEDNTWEPEENLDCPDLIAEFLKKYKKMKEGENNKPREKSESNKRKSNFSNSADDIKSKKKREQSNDIARGFARGLEPERIIGATDSSGELMFLMKWKNSDEADLVPAKEANVKCPQVVISFYEERLTWHSYPEDAENKEKETAKS |
| HP1α-γCDγCSD  (193aa) | MGKKTKRTADSSSSEDEEEFVVEKVLDRRVVNGKVEYFLKWKGFTDADNTWEPEENLDCPELIEAFLNSQKKYKKMKEGENNKPREKSESNKRKSNFSNSADDIKSKKKREQSNDIARGFARGLDPERIIGATDSSGELMFLMKWKDSDEADLVLAKEANMKCPQIVIAFYEERLTWHSCPEDAENKEKETAKS |
| HP1α-CDβ  (190aa) | MGKKTKRTADSSSSEDEEEYVVEKVLDRRVVKGKVEYLLKWKGFSDEDNTWEPEENLDCPDLIAEFLKKYKKMKEGENNKPREKSESNKRKSNFSNSADDIKSKKKREQSNDIARGFERGLEPEKIIGATDSCGDLMFLMKWKDTDEADLVLAKEANVKCPQIVIAFYEERLTWHAYPEDAENKEKETAKS |
| HP1α-CDγ  (193aa) | MGKKTKRTADSSSSEDEEEFVVEKVLDRRVVNGKVEYFLKWKGFTDADNTWEPEENLDCPELIEAFLNSQKKYKKMKEGENNKPREKSESNKRKSNFSNSADDIKSKKKREQSNDIARGFERGLEPEKIIGATDSCGDLMFLMKWKDTDEADLVLAKEANVKCPQIVIAFYEERLTWHAYPEDAENKEKETAKS |
| HP1α-CSDβ  (191aa) | MGKKTKRTADSSSSEDEEEYVVEKVLDRRVVKGQVEYLLKWKGFSEEHNTWEPEKNLDCPELISEFMKKYKKMKEGENNKPREKSESNKRKSNFSNSADDIKSKKKREQSNDIARGFARGLEPERIIGATDSSGELMFLMKWKNSDEADLVPAKEANVKCPQVVISFYEERLTWHSYPEDAENKEKETAKS |
| HP1α-CSDγ  (191aa) | MGKKTKRTADSSSSEDEEEYVVEKVLDRRVVKGQVEYLLKWKGFSEEHNTWEPEKNLDCPELISEFMKKYKKMKEGENNKPREKSESNKRKSNFSNSADDIKSKKKREQSNDIARGFARGLDPERIIGATDSSGELMFLMKWKDSDEADLVLAKEANMKCPQIVIAFYEERLTWHSCPEDAENKEKETAKS |

**b)** Double-stranded DNA sequence used in the simulations.

| 147 bp 601 dsDNA | CTGGAGAATCCCGGTGCCGAGGCCGCTCAATTGGTCGTAGACAGCTCTAGCACCGCTTAAACGCACGTACGCGCTGTCCCCCGCGTTTTAACCGCCAAGGGGATTACTCCCTAGTCTCCAGGCACGTGTCAGATATATACATCCTGT |
| --- | --- |
